# Retracted: Involvement of Nrf2-Mediated Upregulation of Heme Oxygenase-1 in Mollugin-Induced Growth Inhibition and Apoptosis in Human Oral Cancer Cells

**DOI:** 10.1155/2020/1915927

**Published:** 2020-08-16

**Authors:** BioMed Research International

*BioMed Research International* has retracted the article titled “Involvement of Nrf2-Mediated Upregulation of Heme Oxygenase-1 in Mollugin-Induced Growth Inhibition and Apoptosis in Human Oral Cancer Cells” [1]. In February 2020, we were made aware of the results of an institutional investigation which raised concerns with the Western blots shown in Figures 3(a) and 3(b).

We contacted the authors to respond to these concerns however we did not receive a response. With the agreement of the editorial board, this article is being retracted due to concerns with the data.
